# Worse risk profile, number of grafts and hospital death but acceptable late survival in females undergoing coronary surgery: a 20-year propensity matched analysis

**DOI:** 10.1136/openhrt-2025-003894

**Published:** 2026-03-02

**Authors:** Lauren Kari Dixon, Ettorino Di Tommaso, Marco Gemelli, Domenico Vito Bruno, Raimondo Ascione

**Affiliations:** 1 Bristol Heart Institute Hospital, University Hospital Bristol Weston NHS Foundation Trust, Bristol, Somerset, UK; 2 Cardiovascular Medicine, Medical School, Faculty of Health and LIfe Science, University of Bristol, Bristol, UK; 3 Health Services Research and Policy, London School of Hygiene & Tropical Medicine, London, UK; 4 Department of Minimally Invasive Cardiac Surgery, Ospedale Galeazzi - Sant’Ambrogio Centro Cardiotoracico, Milan, Italy

**Keywords:** Coronary Artery Bypass, Coronary Vessels, Outcome Assessment, Health Care

## Abstract

**Objective:**

To evaluate sex differences in perioperative characteristics, in-hospital outcomes and long-term survival following coronary artery bypass grafting (CABG).

**Methods:**

Prospective data were collected for all patients undergoing isolated CABG at a single centre during 2001–2021. Baseline characteristics were adjusted between females and males using 1:1 propensity score matching (nearest-neighbour, without replacement). Kaplan-Meier analysis assessed long-term survival. A predefined sub-analysis assessed risk mitigation associated with using off-pump CABG (OPCABG) in females in the matched cohort.

**Results:**

Prematching, 11 563 males and 2573 females were included. Females were older with higher prevalences of class III–IV angina, hypertension and diabetes. After matching, 2573 patients per group were analysed, with standardised mean differences <0.1 for all covariates. Females had fewer left internal mammary artery (LIMA) grafts (84% vs 88%, p<0.001), fewer total grafts (median 2 vs 3, p<0.001), higher in-hospital mortality (2.2% vs 1.3%, OR 1.74, 95% CI 1.14 to 2.71, p=0.011) and longer hospital stays (median 7 days vs 6 days, beta 0.51, 95% CI 0.12 to 0.90, p=0.01). Long-term survival was similar (stratified log-rank p=0.79). OPCABG mitigated the risk of in-hospital mortality in females (1.1% males vs 1.6% females, OR 0.69, 95% CI 0.33 to 1.43, p=0.32; 1.6% OPCABG females vs 3.0% on-pump females, OR 0.53, 95% CI 0.31 to 0.91, p=0.021).

**Conclusions:**

Females suffer higher in-hospital mortality and receive fewer LIMA and total number of grafts than males; however, 20-year survival is similar. OPCABG protects females from in-hospital mortality. A new female-tailored peri-operative care approach is warranted for females undergoing CABG.

**WHAT IS ALREADY KNOWN ON THIS TOPIC**

- Females undergoing coronary artery bypass grafting (CABG) are known to have worse short-term outcomes than males, including higher in-hospital mortality. However, many studies do not adequately adjust for baseline risk or assess long-term survival, and few evaluate the potential benefits of off-pump CABG (OPCABG) in high-risk female patients.

**WHAT THIS STUDY ADDS**

- In this 20-year propensity-matched analysis, females had higher in-hospital mortality than males despite comparable baseline characteristics, but long-term survival was similar. A predefined subgroup analysis found lower in-hospital mortality in females undergoing OPCABG, suggesting it may mitigate early sex-based disparities in outcomes.

**HOW THIS STUDY MIGHT AFFECT RESEARCH, PRACTICE OR POLICY**

- These findings underscore the need for a sex-specific perioperative strategy for women undergoing CABG. They also support the exploration of OPCABG as a potentially protective strategy for female patients and reinforce the importance of equitable surgical care and guideline-based grafting practices.

## Introduction

 Coronary artery disease (CAD) remains the leading cause of death in women worldwide; however, female-specific peri-operative predictors of early and long-term outcomes after coronary artery bypass grafting (CABG) remain poorly understood.^1^ Women typically present at an older age with more comorbidities, such as hypertension and diabetes, compared with men.^2^ They often experience atypical symptoms leading to diagnostic delays^3^ and are more likely to exhibit microvascular dysfunction and diffuse atherosclerosis.^4^ These sex-based differences in CAD pathophysiology may reduce the effectiveness of existing revascularisation strategies and intraoperative management, potentially causing inequalities in perioperative care and worse outcomes for females undergoing CABG.^5^

Previous studies report higher perioperative mortality, morbidity and longer hospital stays in women than men.^6 7^ While long-term outcomes in females undergoing CABG have been examined,^1^ further research is needed that accounts for baseline risk differences, extended follow-up and evaluates the impact of off-pump CABG (OPCABG), which may benefit high-risk female patients.^8^

This study aimed to assess sex differences in perioperative characteristics, short-term complications and long-term survival following CABG, using 20 years of single-centre data with propensity score matching (PSM) and a predefined OPCABG subgroup analysis.

## Methods

### Selected population and perioperative management

All adult patients undergoing isolated elective or urgent CABG between 2001 and 2021 were included. Exclusions were emergency and salvage cases. The institutional standard CABG protocol can be found in the online supplemental material. Briefly, the decision for CABG surgery was based on Heart Team consensus or following a referral by a senior cardiologist based on coronary angiography confirming severe CAD, echocardiographic left ventricular ejection fraction (LVEF) assessment and, when needed, cardiac MRI. LVEF was categorised as normal (>50%), moderate (30–49%) or poor (<30%).

On the first post-operative day, all patients commenced temporary enoxaparin and long-term antiplatelets, with enoxaparin stopped at discharge. Anaesthetic, perfusion and surgical techniques were based on standardised protocols.^9^,^11^ Heparin was given at a dose of 300 IU/kg to achieve a target activated clotting time (ACT) of ≥400 s before commencement of cardiopulmonary bypass (CPB). For OPCABG, heparin (100 IU/kg) was administered before the start of the first anastomosis to achieve an ACT >300 s. Postoperatively, patients were initially managed in an intensive care unit (ICU) and stepped down to ward-based care and discharged from hospital when fulfilling predefined institutional criteria.

### Study design and data collection

Baseline risk factors, operative details, complications, hospital stay and survival (via Office for National Statistics) were prospectively collected and retrospectively analysed; missing data were hand-searched and retrieved from clinical records to complete the dataset, therefore formal imputation was not required. Follow-up for survival was censored on 31 January 2023. A PSM analysis was planned, followed by a predefined subgroup analysis of only patients having OPCABG. A diagram showing patient selection is shown in the online supplemental figure S1.

### Propensity score matching

PSM was used to balance baseline differences between females and males. Operative variables were excluded from the PSM model, as these may lie on the causal pathway between sex and outcomes and could introduce over-adjustment bias. Propensity scores were calculated using logistic regression, with sex as the dependent variable and age, diabetes, hypertension, smoking, chronic obstructive pulmonary disease (COPD), LVEF, prior myocardial infarction (MI) and atrial fAtrial Fibrillation (AF) as covariates. Definitions were based on standard clinical documentation within our institutional database. Comorbidities were recorded as binary (present/absent) based on clinician diagnosis. Smoking history was defined as any prior or current tobacco use. Detailed data on graft configuration and completeness of revascularisation were not consistently available and were therefore not included in the propensity model. This limitation was addressed by adjusting for all major preoperative and operative variables consistently available in the dataset. Nearest-neighbour matching (1:1) was performed without replacement, using a calliper width of 0.2 SD of the logit of the propensity score. Covariate balance was evaluated using standardised mean differences (SMD), with SMD <0.1 indicating adequate balance.

### Outcome measures

The primary outcome of the study was postoperative in-hospital mortality. Key secondary outcomes included long-term survival, defined as the time from operation to death or follow-up time point, postoperative re-operation for bleeding, haemofiltration/dialysis, stroke, deep sternal wound infection/mediastinitis and length of hospital stay.

### Statistical analysis

Normality was tested by Shapiro-Wilk test. Normally distributed data are presented as mean and SD and compared by Student’s t-test; non-normal data as median and IQR and compared by Wilcoxon signed-rank or rank-sum tests, depending on matching status. Categorical variables were compared by χ^2^, Fisher’s exact, Wilcoxon rank sum or McNemar’s tests as appropriate. For comparisons in the propensity-matched cohort, statistical tests appropriate for paired data were used. Logistic and linear regressions were used to assess associations between sex and outcomes. Kaplan-Meier curves, log-rank (pre-matched) and stratified log-rank (matched) tests compared survival. Kaplan-Meier curves were truncated at the point where 10% of the study population remained at risk. A logistic regression model including an interaction term between sex and use of CPB (on-pump vs off-pump) was fitted to assess whether OPCABG modified the association between sex and in-hospital mortality. Statistical analyses were performed using R (V.4.0.5), with the *MatchIt* package, the *survival* and the *gtsummary* package. A two-tailed p value of <0.05 was considered significant.

## Results

### Baseline characteristics of the pre-matched population

A total of 14 136 consecutive patients were included in the study. Of these, 11 563 (81.9%) were male and 2573 (18.1%) were female. The baseline characteristics of the pre-matched population are shown in table 1. Females were older (median 71 years vs 68 years, p<0.001) and had higher rates of diabetes (26% vs 22%, p<0.001) and hypertension (77% vs 73%, p<0.001). Severe left main stem disease was more frequent in females, but this did not reach statistical significance (24.5% males vs 22.6% females, p=0.072), with both groups having a median of three diseased vessels (p=0.4). Females more often required urgent surgery (51% vs 48%, p<0.001), presented with worse Canadian Cardiovascular Society (CCS) angina scores but better preserved LVEF (78% vs 73%, p<0.001). No significant differences were found in COPD (p>0.9) or AF (p=0.3), while smoking prevalence was lower in females (52% vs 71%, p<0.001).

**Table 1:** Baseline characteristics of the pre-matched population

Characteristic	Males N=11 563*	Females N=2573*	P value†
Age (years)	68 (61, 74)	71 (63, 76)	<0.001
Left main stem disease	2877 (24.5%)	591 (22.6%)	0.072
Diabetes mellitus	2569 (22%)	672 (26%)	<0.001
Hypertension	8429 (73%)	1990 (77%)	<0.001
COPD	1025 (8.9%)	230 (8.9%)	>0.9
Urgency			<0.001
Elective	6033 (52%)	1249 (49%)	
Urgent	5530 (48%)	1324 (51%)	
BMI	28.4 (25.6, 30.2)	28.6 (25.0, 31.2)	0.8
AF	542 (4.7%)	132 (5.1%)	0.3
Previous MI	5399 (47%)	1095 (43%)	<0.001
Smoking history	8162 (71%)	1361 (52%)	<0.001
Previous stroke	412 (3.6%)	92 (3.6%)	>0.9
LVEF			<0.001
Good (LVEF ≥50%)	8447 (73%)	2003 (78%)	
Moderate (LVEF 30–49%)	2475 (21%)	450 (17%)	
Poor (LVEF <30%)	641 (5.5%)	120 (4.7%)	
Number of diseased coronary vessels	3 (2,3)	3 (2, 3)	0.4
CCS score			<0.001
0	1604 (14%)	362 (14%)	
1	1329 (11%)	214 (8.3%)	
2	4134 (36%)	767 (30%)	
3	2729 (24%)	700 (27%)	
4	1767 (15%)	530 (21%)	

* Median (Q1, Q3); n (%).

† Wilcoxon rank sum test; Pearson’s χ2 test; Fisher’s exact test.

AF, atrial fibrillation; BMI, body mass index; CCS, Canadian Cardiovascular Society; COPD, chronic obstructive pulmonary disease; LVEF, left ventricular ejection fraction; MI, myocardial infarction.

### Health outcome of the pre-matched population

The use of CABG surgery with CPB was 55% in both groups with no differences in CPB time. Females received fewer left internal mammary artery (LIMA) grafts (84% vs 91%, p<0.001) and fewer total grafts (median 2 vs 3, p<0.001) (online supplemental table S1). Postoperatively, females had higher in-hospital mortality (2.2% vs 1.4%, OR 1.62, 95% CI 1.19 to 2.20, p=0.002), longer hospital stays (median 7 vs 6 days, beta 0.71, 95% CI 0.40 to 1.0, p<0.001) and higher stroke rates (1.1% vs 0.7%, OR 1.55, 95% CI 1.01 to 2.38, p=0.046). Rates of deep sternal wound infection, re-operation for bleeding and haemofiltration/dialysis were similar (online supplemental table S2). Over 20 years, females had poorer survival than males (log-rank p<0.001) (online supplemental figure S2).

### Baseline characteristics of the post-matched population

After PSM, 2573 males and 2573 females formed the matched cohort. SMDs were <0.1 for all variables, indicating no residual baseline differences (table 2).

**Table 2:** Baseline characteristics of the matched population

Characteristic	Males N=2573*	Females N=2573*	P value†	SMD
Age (years)	70 (63, 76)	71 (63, 76)	0.5	0.0029
Left main stem disease	610 (23%)	601 (22%)	0.6	−0.0161
Diabetes mellitus	661 (26%)	672 (26%)	0.7	0.0097
Hypertension	1992 (77%)	1990 (77%)	>0.9	−0.0019
COPD	233 (9.1%)	230 (8.9%)	0.9	−0.0041
Urgency			0.8	
Elective	1259 (49%)	1249 (49%)		−0.0078
Urgent	1314 (51%)	1324 (51%)		0.0078
BMI	28.3 (25.5, 29.9)	28.6 (25.0, 31.2)	0.12	−0.0163
AF	126 (4.9%)	132 (5.1%)	0.7	0.0106
Previous MI	1120 (44%)	1095 (43%)	0.5	−0.0197
Smoking history	1373 (53%)	1361 (52%)	0.6	0.0079
Previous stroke	103 (4.0%)	92 (3.6%)	0.9	−0.0166
LVEF			>0.9	
Good (LVEF≥50%)	2002 (78%)	2003 (78%)		0.0009
Moderate (LVEF 30–49%)	446 (17%)	450 (17%)		0.0041
Poor (LVEF<30%)	125 (4.9%)	120 (4.7%)		−0.0092
Number of diseased coronary vessels	3 (2, 3)	3 (2, 3)	0.7	−0.0286
CCS score			0.73	0.008
0	365 (14%)	362 (14%)		
1	232 (9%)	214 (8%)		
2	772 (30%)	767 (30%)		
3	648 (25%)	700 (27%)		
4	556 (22%)	530 (21%)		

* Median (Q1, Q3); n (%).

† Wilcoxon signed-rank test; McNemar’s test.

AF, atrial fibrillation; BMI, body mass index; CCS, Canadian Cardiovascular Society; COPD, chronic obstructive pulmonary disease; LVEF, left ventricular ejection fraction; MI, myocardial infarction; SMD, standardised mean difference.

### Intraoperative characteristics of the matched population

Females were less likely to receive a LIMA graft (84% vs 88%) or a right internal mammary artery (RIMA) graft (2.1% vs 4.1%) and had a lower median number of grafts (2 vs 3) compared with males. Use of CPB was similar between groups (females 55% vs males 57%), although CPB time showed a moderate imbalance (median 55 min vs 60 min, SMD 0.4) despite a non-significant p value (p=0.2) (table 3).

**Table 3:** Operative characteristics of the matched population

Characteristic	Males N=2573*	Females N=2573*	P value*†*	SMD
CPB used	1474 (57%)	1421 (55%)	0.14	0.04
CPB time‡	90 (60, 107)	86 (55, 105)	0.2	0.4
LIMA graft	2275 (88%)	2168 (84%)	<0.001	0.12
RIMA graft	105 (4.1%)	53 (2.1%)	<0.001	0.12
BIMA graft	87 (3.4%)	42 (1.6%)	<0.001	0.17
Radial artery graft	282 (11%)	248 (9.6%)	0.12	0.04
Number of grafts	3 (2, 3)	2 (2, 3)	<0.001	0.18

* n (%); median (Q1, Q3).

† McNemar’s test; Wilcoxon signed-rank test.

‡ Off-pump CABG excluded.

BIMA, bilateral internal mammary arteries; CABG, coronary artery bypass grafting; CPB, cardiopulmonary bypass; LIMA, left internal mammary artery; RIMA, right internal mammary artery; SMD, standardised mean difference.

### In-hospital outcomes of matched population

After matching, females continued to have higher in-hospital mortality than males (2.2% vs 1.3%, OR 1.74, 95% CI 1.14 to 2.71, p=0.011) and longer postoperative hospital stays (median 7 days vs 6 days, OR 0.51, 95% CI 0.12 to 0.90, p=0.01). Females had lower rates of re-operation for bleeding (2.5% vs 3.5%, OR 0.69, 95% CI 0.50 to 0.94, p=0.02). No significant differences were found for deep sternal wound infection (1.3% vs 1.4%, p=0.5), stroke (1.1% vs 0.9%, p=0.56) or need for haemofiltration/dialysis (1.0% in both groups, p>0.9) (table 4).

**Table 4:** Short-term outcomes of the matched population

Outcome	Males N=2573*	Females N=2573*	OR†	95% CI	P value
In-hospital mortality	33 (1.3%)	57 (2.2%)	1.74	1.14 to 2.71	0.011
Postoperative stroke	24 (0.9%)	28 (1.1%)	1.17	0.69 to 1.99	0.56
Re-operation for bleeding	90 (3.5%)	63 (2.5 %)	0.69	0.50 to 0.94	0.02
Postoperative dialysis	27 (1.0%)	27 (1.0%)	1.00	0.59 to 1.70	>0.9
Deep sternal wound infection	36 (1.4%)	33 (1.3%)	1.09	0.69 to 1.72	0.5
Length of hospital stay	6 (5, 9)	7 (6, 10)	0.51	0.12 to 0.90	0.01

* n (%); median (Q1, Q3).

† OR/beta co-efficient.

### Long-term survival of the matched population

**Figure 1:**
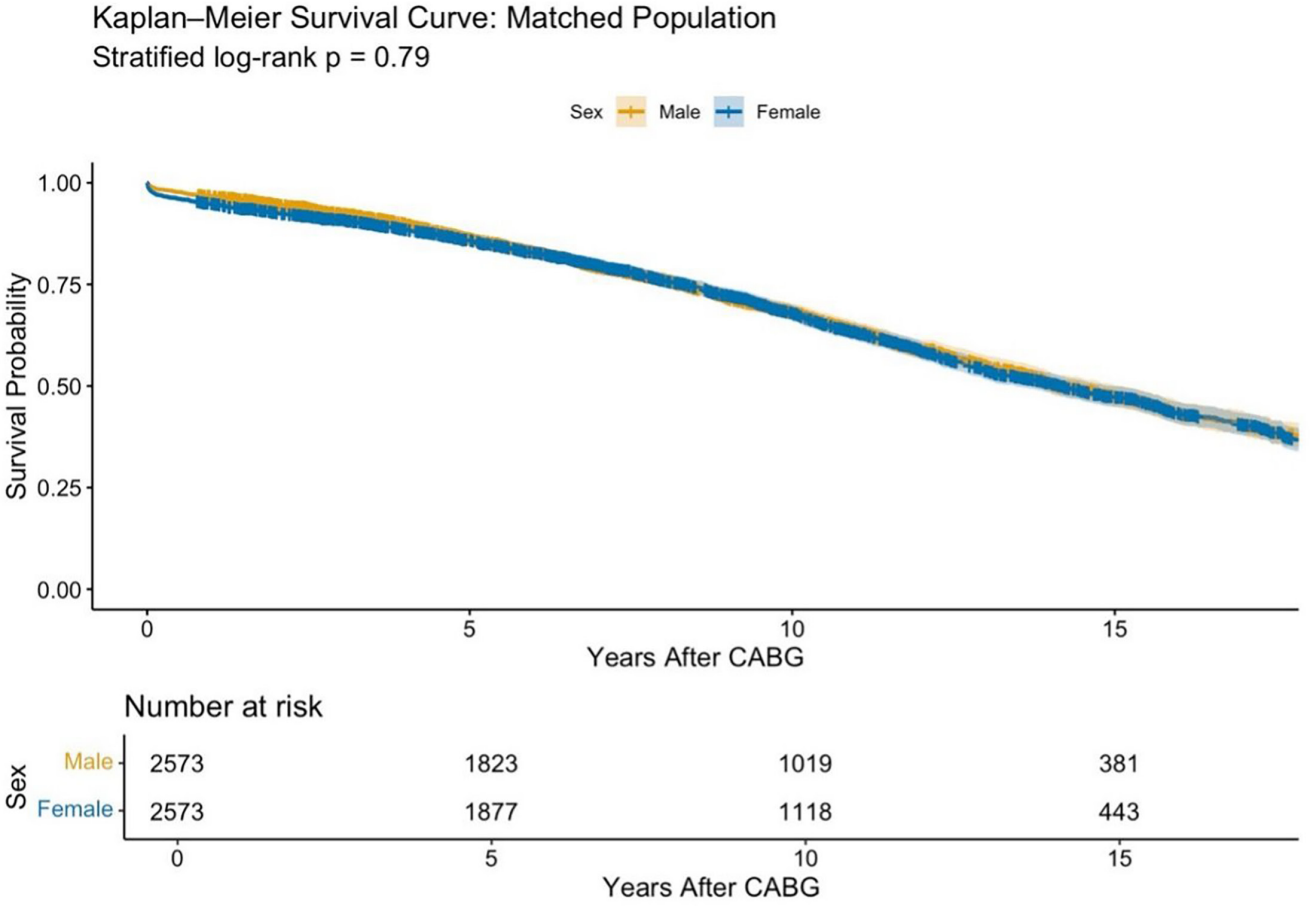
Kaplan-Meier survival analysis of the matched population, by sex. CABG, coronary artery bypass grafting.

Median follow-up was 8.3 years (IQR 4.5–12.4) for males and 9.0 years (IQR 4.7–13.2) for females. Kaplan-Meier analysis showed no significant difference in 20-year survival between groups (log-rank p=0.79; figure 1), with survival curves closely aligned, indicating similar long-term mortality risks. This was confirmed by a supplementary landmark survival analysis in the matched population, excluding patients suffering in-hospital death (stratified log-rank p=0.65; online supplemental figure S3).

### Predefined subgroup analysis to ascertain the impact of OPCABG surgery

Among the matched cohort, 1099 males and 1152 females underwent OPCABG and were included in this subgroup analysis. Baseline characteristics were comparable, including age (median 69 vs 70 years, p>0.9), diabetes (26% vs 25%, p=0.6), hypertension (79% vs 78%, p=0.6), severe left main stem disease (22% vs 25%, p=0.4) and prior MI (46% vs 45%, p=0.7) (online supplemental table S3). Females had a higher BMI (28.6 vs 28.1, p=0.04), while other variables such as urgency of surgery, LVEF and smoking history did not differ between subgroups (all p>0.05).

Intraoperatively, females received fewer grafts (median 2 vs 3, p<0.001) and fewer LIMA grafts (91% vs 94%, p=0.015) (online supplemental table S4), though LIMA usage was higher than in on-pump CABG patients. Postoperatively, no significant sex differences were observed for in-hospital mortality (1.6% vs 1.1%, OR 0.69, 95% CI 0.33 to 1.43, p=0.32), re-operation for bleeding, stroke, dialysis or sternal infections (all p>0.05) (online supplemental table S5). Females had longer hospital stays (median 7 days vs 6 days, beta 0.50, 95% CI 0.25 to 0.75, p<0.001).

Comparing females undergoing OPCABG to those receiving on-pump CABG, in-hospital mortality was significantly lower in OPCABG (1.6% vs 3.0%, OR 0.53, 95% CI 0.31 to 0.91, p=0.021). Median follow-up was 10.6 years (IQR 6.8–13.7) for males and 10.9 years (IQR 7.3–14.4) for females. Long-term survival was similar (stratified log-rank p=0.85; online supplemental figure S4).

## Discussion

In this study, females undergoing CABG presented with a worse risk profile, including more comorbidities and adverse preoperative characteristics. In addition, they experienced intraoperative inequalities with fewer LIMA grafts and overall number of grafts. Despite PSM to balance for these preoperative differences, females suffered higher in-hospital mortality than males; however, the long-term survival was comparable.

Others have reported that females suffer higher in-hospital mortality following CABG,^2^,^7^ even after adjusting for baseline differences. Noticeably, data on the long-term survival between sexes following CABG have been controversial.^7 8 12^ The present study confirms that females experience higher in-hospital mortality than males even after PSM. While this finding may indicate a genuine predisposition to worse in-hospital outcome associated with being female, more studies are needed in this area to ascertain if females might benefit from a female-specific per-operative approach.

This study highlights that females experienced intraoperative inequalities, including fewer LIMA grafts and overall number of grafts than males despite presenting with a worse CCS angina score and a similar number of diseased coronary arteries. The LIMA graft represents the gold standard for coronary revascularisation given its superior long-term patency rate versus other conduits.^13^ Its reduced use in females may reflect differences in surgical strategies or worse intraoperative anatomical findings. Others have found that females tend to receive fewer grafts.^14 15^ The relatively low LIMA usage observed in our cohort, particularly among females, may partly reflect historical practice patterns or patient-specific factors, although RIMA and radial arteries were used on average in 3% and 10.5% of the cases, hence resulting in a much higher rate of patient receiving at least one arterial graft. The difference in LIMA usage across groups within the same institution warrants reflections as a potential contributor to outcome differences and therefore a target for quality improvement. Reassuringly, the ongoing ROMA:Women trial is expected to provide prospective, sex-specific data on conduit selection and outcomes in female CABG patients, helping to address some of these persistent disparities.^16^

It might be argued that these results are due to female-specific predisposition. Non-obstructive CAD and microvascular dysfunction are more common in females,^17 18^ which might affect decision-making for revascularisation strategies. It has also been suggested that narrower coronary arteries and small diameters of grafting conduits may worsen outcomes in females due to increased risk of occlusion or thrombosis.^3^ This difference persisted in the on-pump subgroup, suggesting that reduced graft numbers in females are more likely related to coronary anatomy and disease distribution than to technical constraints imposed by off-pump surgery. Together, these findings indicate that females undergoing CABG may benefit from a more female-specific approach, such as autologous conduit harvesting, intraoperative checks of blood flow across the anastomosis and/or more aggressive antiplatelet therapy in the early postoperative period.^19^,^21^

The relatively high use of OPCABG reflects centre-specific long-term expertise and practice patterns over the study period and should be interpreted in this context.^11 22 23^ The predefined sub-analysis of the matched OPCABG population of the present study suggests that OPCABG may mitigate some risk of in-hospital mortality in females. This is supported by similar mortality between females and males in that sub-analysis and by the observation that the in-hospital mortality for OPCABG females was lower than that observed in the female CABG cohort. While an interaction analysis did not demonstrate a statistically significant interaction between sex and OPCABG with respect to in-hospital mortality, the subgroup findings remain hypothesis-generating and warrant further investigation in larger or multicentre datasets. Prior studies report that OPCABG surgery may reduce the post-operative stroke and renal dysfunction rates,^1122^,^24^ especially in selected female patients.^25^

Females experienced fewer re-operations for bleeding than males, despite presenting with worse pre-operative risk profiles. This finding contrasts with some prior studies reporting more transfusions in females after CABG, highlighting the heterogeneity of the existing literature.^6 26^ Re-operation for bleeding is influenced by multiple factors beyond patient sex, including surgical technique, perioperative anticoagulation and transfusion protocols and institutional thresholds for re-exploration. In our propensity-matched cohort, key metabolic and clinical risk factors were balanced, and absolute event rates were low, which may partly explain the observed differences. The smaller coronary vasculature and more diffuse coronary disease seen in females may trigger different haemodynamic or molecular responses that might influence early or late health outcomes such as fewer re-openings for bleeding, but this speculation warrants further study. Moreover, females may benefit from female-tailored thresholds for re-operations or postoperative care. There are obvious hormonal differences between sexes; for example, the protective effects of oestrogen in premenopausal women play a key role in haemostasis.^27^ In addition, oestrogen has vaso-protective effects and may influence platelet aggregation, clot formation and haemostasis. These effects may explain the lower rates of postoperative re-opening for bleeding observed in females in this study. However, postmenopausal women experiencing a decline in oestrogen levels may not exhibit these protective effects, and it remains unclear whether hormonal status influences bleeding risks in post-menopausal women. Recently, the REBOOT trial highlighted sex-specific treatment effects, showing that women had worse outcomes when treated with β-blockers after myocardial infarction, unlike men, suggesting that female patients may respond differently to standard cardiovascular therapies.^28^ Further research is needed in this important area to help females undergoing CABG or any major surgery.

Female sex is reported as a risk factor for sternal wound complications;^29^ however, in our study, a difference in the rate of deep sternal wound infection was not found. An explanation for this may be that other important risk factors were well balanced within the PSM model. In addition, our institution routinely applies a structured pre-operative risk stratification and prevention pathway for sternal wound infection, with targeted postoperative measure in women, a sternal support bra.^30^

Despite a worse preoperative risk profile and worse in-hospital outcomes, the long-term survival in females was comparable to that of males after matching. This finding was observed in the matched population and in the predefined OPCABG sub-analysis. In addition, this finding was consistent when excluding all in-hospital deaths. This may suggest that, while presenting with worse risk profile and suffering worse in-hospital outcomes, females benefit from an innate long-term resilience as they appeared to do as well as males after discharge. This is in keeping with previous work suggesting that females may experience similar long-term survival compared with males^14 31^ and that with more diffuse coronary disease, females benefit from a more stable disease course over time.^17^ A pooled individual patient-level analysis of four randomised controlled trials found no significant difference in 5-year all-cause mortality between sexes (adjusted HR 1.03, 95% CI 0.94 to 1.14),^32^ despite higher peri-operative risk in females. These findings reinforce the notion that, although females may experience worse early outcomes, they derive comparable long-term survival benefit from CABG when appropriately selected and managed.

The key methodological strengths of this study include the large consecutive cohort, long duration of follow-up and robust PSM achieving excellent covariate balance. There are also limitations to highlight. Despite matching, residual confounding cannot be ruled out. Other clinically important variables such as anaemia and renal function were not consistently available across the entire study period and therefore could not be reliably included in the analysis. Additionally, this was a single centre study, limiting generalisability. However, a large population of consecutive patients was used with only emergency/salvage cases being excluded. Detailed data on graft configuration and completeness of revascularisation were not consistently available and therefore could not be included in the propensity model. This represents a limitation of the present analysis, as these factors are known to influence long-term outcomes. However, given the size and balance of the matched cohort, our results remain informative regarding overall sex-based trends in operative management and survival following CABG. Finally, other important outcomes were not measured, such as quality of life, functional status, need for hospital readmission or repeat revascularisation.

## Conclusion

This study suggests that females undergoing CABG surgery have worse preoperative risk profile, experience some intraoperative inequalities including less LIMA and overall number of grafts, suffer higher in-hospital mortality and longer hospital stay than males. The use of OPCABG surgery may mitigate the risk of in-hospital mortality in females. Despite their worse risk profile, females may benefit from an innate long-term resilience as their long-term survival was similar to males. More could be done to identify and implement a more female-tailored approach for females undergoing CABG surgery.

## Supplementary material

10.1136/openhrt-2025-003894 online supplemental file 1

## Data Availability

Data are available upon reasonable request.
