# Supplementary material for: Worse risk profile, number of grafts and hospital death but acceptable late survival in females undergoing coronary surgery: a 20-year propensity matched analysis
Source: Open Heart. 2026 Mar 2;13(1):e003894. doi: 10.1136/openhrt-2025-003894 (PMC12959041; doi:10.1136/openhrt-2025-003894)
Supplement: online supplemental file 1 [file openhrt-13-1-s001.docx]

**Worse risk profile, number of grafts and hospital death but acceptable late survival in females undergoing coronary surgery: a 20-year propensity matched analysis - Supplementary Material**

Supplementary Methods: Operative protocol for CABG

Pre-operatively, all patients with severe coronary artery disease (CAD) were on routine medications including platelet inhibitors, anti-anginal, statins, β-blockers, diuretics, antihypertensives, angiotensin-converting enzyme inhibitors, calcium channel blockers or hypoglycaemic medications at discretion of their referring cardiologists.

Decision for CABG surgery was based on Heart Team consensus or following a referral by a senior cardiologist based on coronary angiography confirming severe CAD, echocardiographic left ventricular ejection fraction (LVEF) assessment, and, when needed, cardiac magnetic resonance imaging. LVEF was categorized as normal (>50%), moderate (30–49%), or poor (<30%).

On the first post-operative day, all patients commenced temporary enoxaparin and long-term antiplatelets, with enoxaparin stopped at discharge. All pre-operative drugs were restarted over the first 2-3 days after surgery based on monitoring of blood pressure and renal function.

Anaesthetic, perfusion and surgical techniques were based on standardised protocols. Briefly, anaesthesia was induced with midazolam, propofol, and fentanyl 5‐10 mcg/kg, and muscle relaxation was achieved with vecuronium or rocuronium. Mechanical ventilation was guided by a positive end‐expiratory pressure of 5cmH_2_O and normal volume-control ventilation at the following settings: tidal volume of 6-8mL/kg, inspiratory/expiratory ratio of 1:2, FiO_2_ of 100% for 5 minutes to be reduced to 50% thereafter, and ventilation rate of 12/minute aiming to obtain a favourable PaCO_2_ (35.0-45.0mmHg) and pH (7.35–7.45). Heparin was given at a dose of 300IU/kg to achieve a target activated clotting time (ACT) of ≥400 seconds before commencement of cardiopulmonary bypass (CPB). For OPCABG, heparin (100IU/kg) was administered before the start of the first anastomosis to achieve an ACT >300 seconds. Following surgery, patients were ventilated with 60% oxygen using volume-controlled ventilation on the intensive care unit . The extubation process followed predefined protocols based on the presence of systemic temperature of 36°C, a systolic blood pressure >100mmHg, pulse rate <100bpm, and a decreasing blood loss <100mL/h from chest drains.

Table S1: Operative characteristics of the pre-matched population

Table S2: Short-term outcomes of the pre-matched population

Table S3: Subgroup analysis: Baseline characteristics of the matched population, limited to OPCABG

Table S4: Subgroup analysis: Operative characteristics of the matched population, limited to OPCABG

Table S5: Subgroup analysis: Short-term outcomes of the matched population, limited to OPCABG

Figure S1: Diagram of populations used in the study

Figure S2: Kaplan-Meier survival analysis of the pre-matched population, by sex

Figure S3: Kaplan-Meier survival analysis of the matched population with any in-hospital mortality patients excluded, by sex

Figure S4: Subgroup analysis: Kaplan-Meier survival analysis of the matched population, by sex, limited to OPCABG

Table S1: Operative characteristics of the pre-matched population

| **Characteristic** | Males N = 11,563*^1^* | Females N = 2,573*^1^* | **p-value***^2^* |
| --- | --- | --- | --- |
| CPB used | 6,344 (55%) | 1,421 (55%) | 0.7 |
| CPB time* | 87 (57, 102) | 86 (55, 105) | 0.9 |
| LIMA graft | 10,476 (91%) | 2,168 (84%) | <0.001 |
| RIMA graft | 587 (5.1%) | 53 (2.1%) | <0.001 |
| BIMA graft | 531 (4.6%) | 42 (1.6%) | <0.001 |
| Radial artery graft | 1,634 (14%) | 248 (9.6%) | <0.001 |
| Number of grafts | 3 (2,3) | 2 (2,3) | <0.001 |
| *^1^* n (%); Median (Q1, Q3)  *^2^* Pearson’s Chi-squared test; Wilcoxon rank sum test  *Off-pump CABG excluded  *CPB – cardiopulmonary bypass; LIMA – left internal mammary artery; RIMA – right internal mammary artery; BIMA – bilateral internal mammary arteries* | | | |
|  | | | |

Table S2: Short-term outcomes of the pre-matched population

| **Outcome** | Males N = 11,563*^1^* | Females N = 2,573*^1^* | OR^2^ | | 95% CI^3^ | **p-value** |
| --- | --- | --- | --- | --- | --- | --- |
| In-hospital mortality | 156 (1.4%) | 57 (2.2%) | **1.62** | | 1.19 – 2.20 | **0.002** |
| Deep sternal wound infection | 185 (1.6%) | 33 (1.3%) | **0.81** | | 0.56 – 1.17 | 0.26 |
| Re-operation for bleeding | 358 (3.1%) | 63 (2.5 %) | **0.80** | | 0.61 – 1.05 | 0.11 |
| Length of hospital stay | 6 (5, 9) | 7 (6, 10) | 0.71 | | 0.40 - 1.0 | <0.001 |
| Post-operative stroke | 81 (0.7%) | 28 (1.1%) | **1.55** | | 1.01 – 2.38 | **0.046** |
| Post-operative dialysis | 123 (1.1%) | 27 (1.0%) | **0.93** | | 0.61 – 1.41 | 0.73 |
| *^1^* n (%); Median (Q1, Q3)  *^2^* Odds Ratio / beta  ^3^ 95% Confidence Intervals | | | |  | |  |

Table S3: Subgroup analysis: Baseline characteristics of the matched population, limited to OPCABG

| **Characteristic** | Males N = 1,099*^1^* | Females N = 1,152*^1^* | **p-value***^2^* | **SMD** | |  |
| --- | --- | --- | --- | --- | --- | --- |
| Age (years) | 69 (62, 75) | 70 (62, 75) | >0.9 | 0.4 | |  |
| Left main stem disease | 245 (22%) | 285 (25%) | 0.4 | 0.07 | |  |
| Diabetes mellitus | 285 (26%) | 289 (25%) | 0.6 | 0.85 | |  |
| Hypertension | 864 (79%) | 895 (78%) | 0.6 | 0.93 | |  |
| COPD | 96 (8.7%) | 85 (7.4%) | 0.2 | 0.05 | |  |
| Urgency |  |  | 0.9 | 0.01 | |  |
| 1. Elective | 551 (50%) | 575 (50%) |  |  | |  |
| 2. Urgent | 548 (50%) | 577 (50%) |  |  | |  |
| BMI | 28.1 (25.5, 29.7) | 28.6 (25.1, 30.9) | 0.04 | -0.21 | |  |
| AF | 45 (4.1%) | 40 (3.5%) | 0.4 | 0.62 | |  |
| Previous MI | 506 (46%) | 521 (45%) | 0.7 | 0.82 | |  |
| Smoking history | 629 (57%) | 655 (56%) | 0.4 | 0.06 | |  |
| Previous stroke | 41 (3.7%) | 44 (3.8%) | 0.9 | 0.03 | |  |
| LVEF |  |  | 0.9 | 0.02 | |  |
| 1. Good (LVEF >= 50%) | 855 (78%) | 903 (78%) |  |  | |  |
| 2. Moderate (LVEF 30-49%) | 188 (17%) | 195 (17%) |  |  | |  |
| 3.Poor (LVEF < 30%) | 56 (5.1%) | 54 (4.7%) |  |  | |  |
| Number of diseased coronary vessels | 3 (2, 3) | 3 (2, 3) | 0.7 | 0.02 | |  |
| *^1^ Median (Q1, Q3); n (%)* | | | | |  | |
| *^2^ Wilcoxon signed-rank test; McNemar’s test*  *COPD – chronic obstructive pulmonary disease; BMI – body mass index; AF – atrial fibrillation; MI – myocardial infarction; LVEF – left ventricular ejection fraction; CCS – Canadian Cardiovascular Society* | | | | |  | |
|  |  |  |  |  | |  |

Table S4: Subgroup analysis: Operative characteristics of the matched population, limited to OPCABG

| **Characteristic** | Males N = 1,099*^1^* | Females N = 1,152*^1^* | **p-value***^2^* | **SMD** | |  |
| --- | --- | --- | --- | --- | --- | --- |
| LIMA graft | 1,029 (94%) | 1,047 (91%) | 0.015 | 0.1 | |  |
| RIMA graft | 55 (5.0%) | 25 (2.2%) | <0.001 | 0.15 | |  |
| BIMA graft | 47 (4.3%) | 19 (1.6%) | <0.001 | 0.26 | |  |
| Radial artery graft | 191 (17%) | 175 (15%) | 0.2 | 0.06 | |  |
| Number of grafts | 3.00 (2.00, 3.00) | 2.00 (2.00, 3.00) | <0.001 | 0.16 | |  |
| *^1^* n (%); Median (Q1, Q3)  *2 McNemar's test; Wilcoxon signed-rank test*  *LIMA – left internal mammary artery;1 RIMA – right internal mammary artery; BIMA – bilateral internal mammary arteries* | | | | |  | |
|  | | | | |  | |

Table S5: Subgroup analysis: Short-term outcomes of the matched population, limited to OPCABG

| **Outcome** | Males N = 1,099*^1^* | Females N = 1,152*^1^* | OR^2^ | | 95% CI^3^ | **p-value** |
| --- | --- | --- | --- | --- | --- | --- |
| In-hospital mortality | 12 (1.1%) | 19 (1.6%) | 0.69 | | 0.33 - 1.43 | 0.32 |
| Deep sternal wound infection | 0 (0%) | 0 (0%) |  | |  |  |
| Re-operation for bleeding | 18 (1.6%) | 27 (2.3%) | 0.68 | | 0.37 - 1.24 | 0.21 |
| Length of hospital stay | 6.0 (5.0, 8.0) | 7.0 (5.0, 9.0) | **0.50** | | **0.25 - 0.75** | **<0.001** |
| Post-operative stroke | 6 (0.6%) | 5 (0.4%) | 1.38 | | 0.42 - 4.55 | 0.77 |
| Post-operative dialysis | 8 (0.7%) | 13 (1.1%) | 0.64 | | 0.26 - 1.55 | 0.32 |
| *^1^* n (%); Median (Q1, Q3)  *^2^* Odds Ratio / beta co-efficient  ^3^ 95% Confidence Intervals | | | |  | |  |


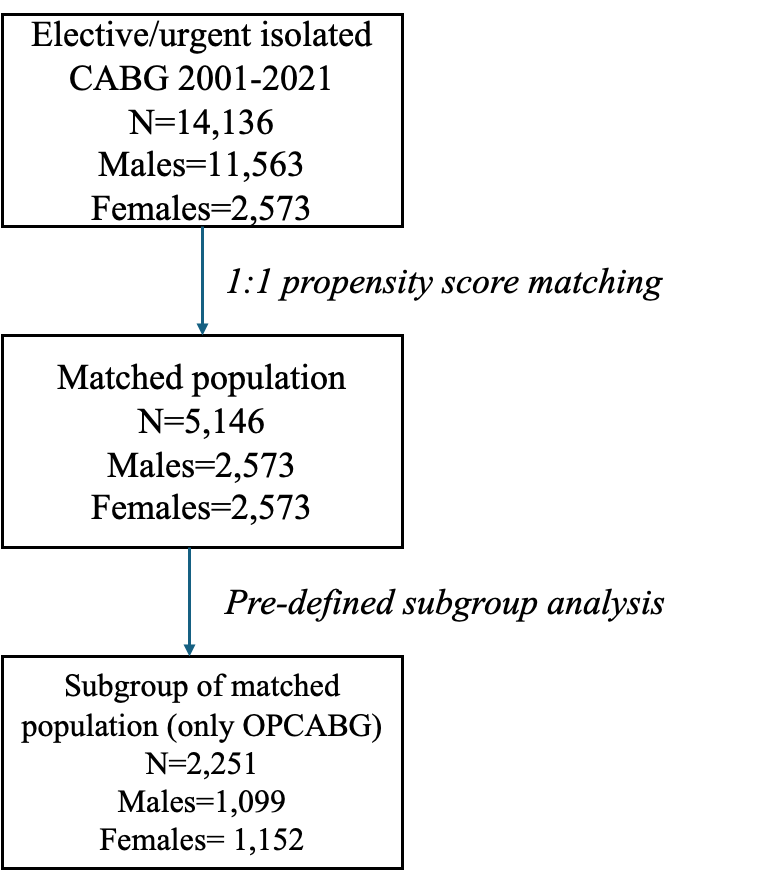


Figure S1: Diagram of populations used in the study


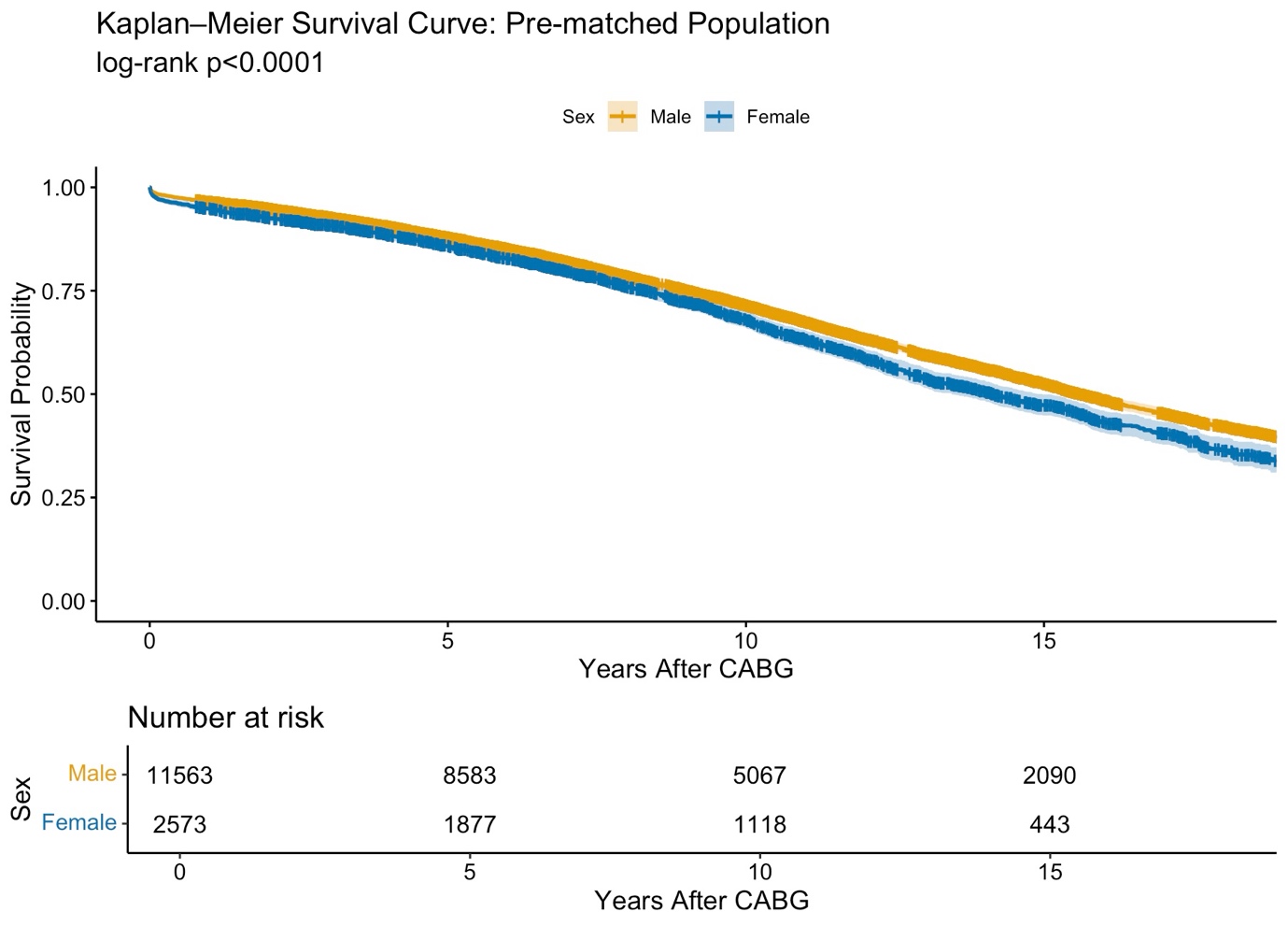


Figure S2: Kaplan-Meier survival analysis of the pre-matched population, by sex


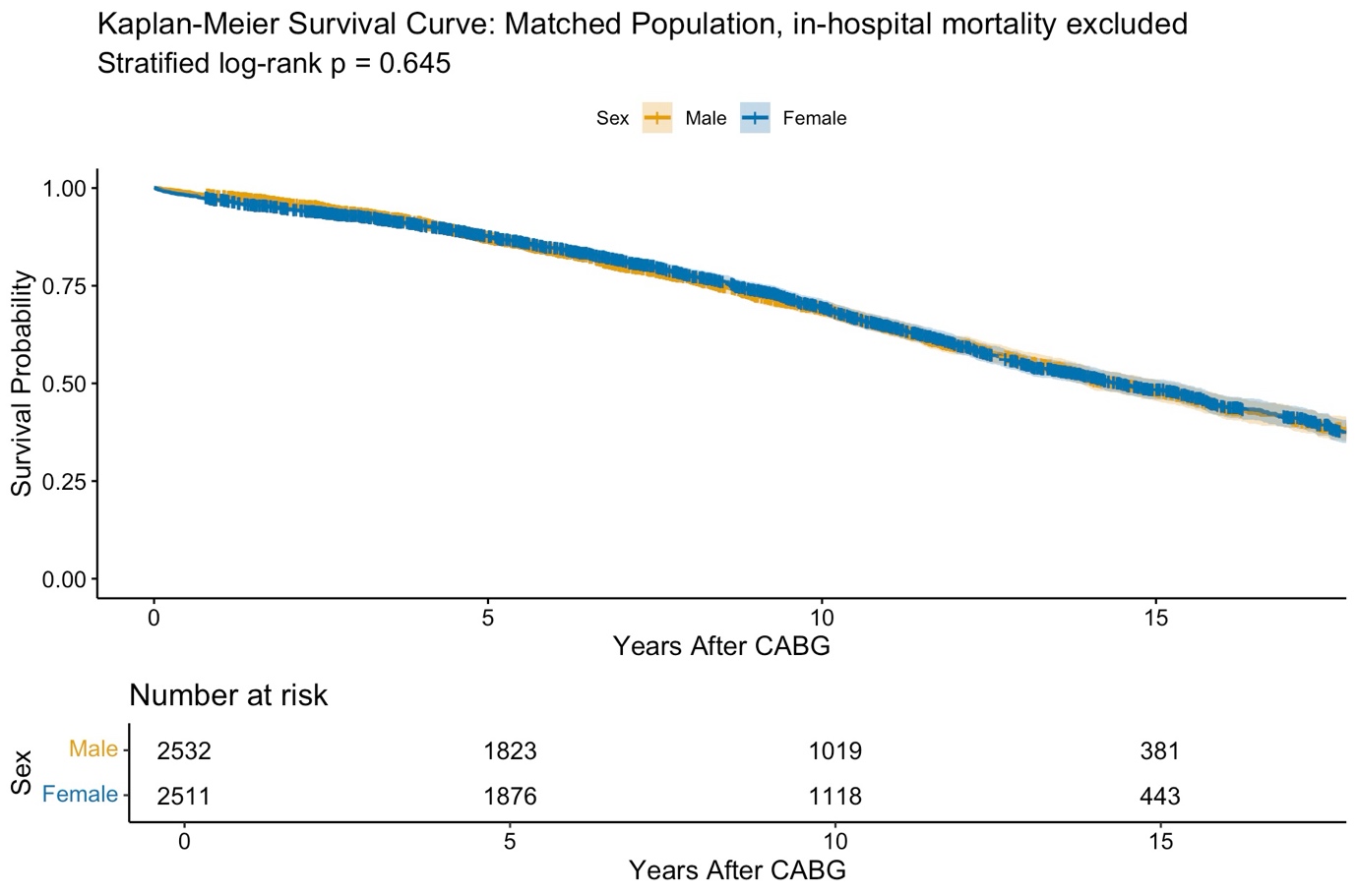


Figure S3: Kaplan-Meier survival analysis of the matched population with any in-hospital mortality patients excluded, by sex


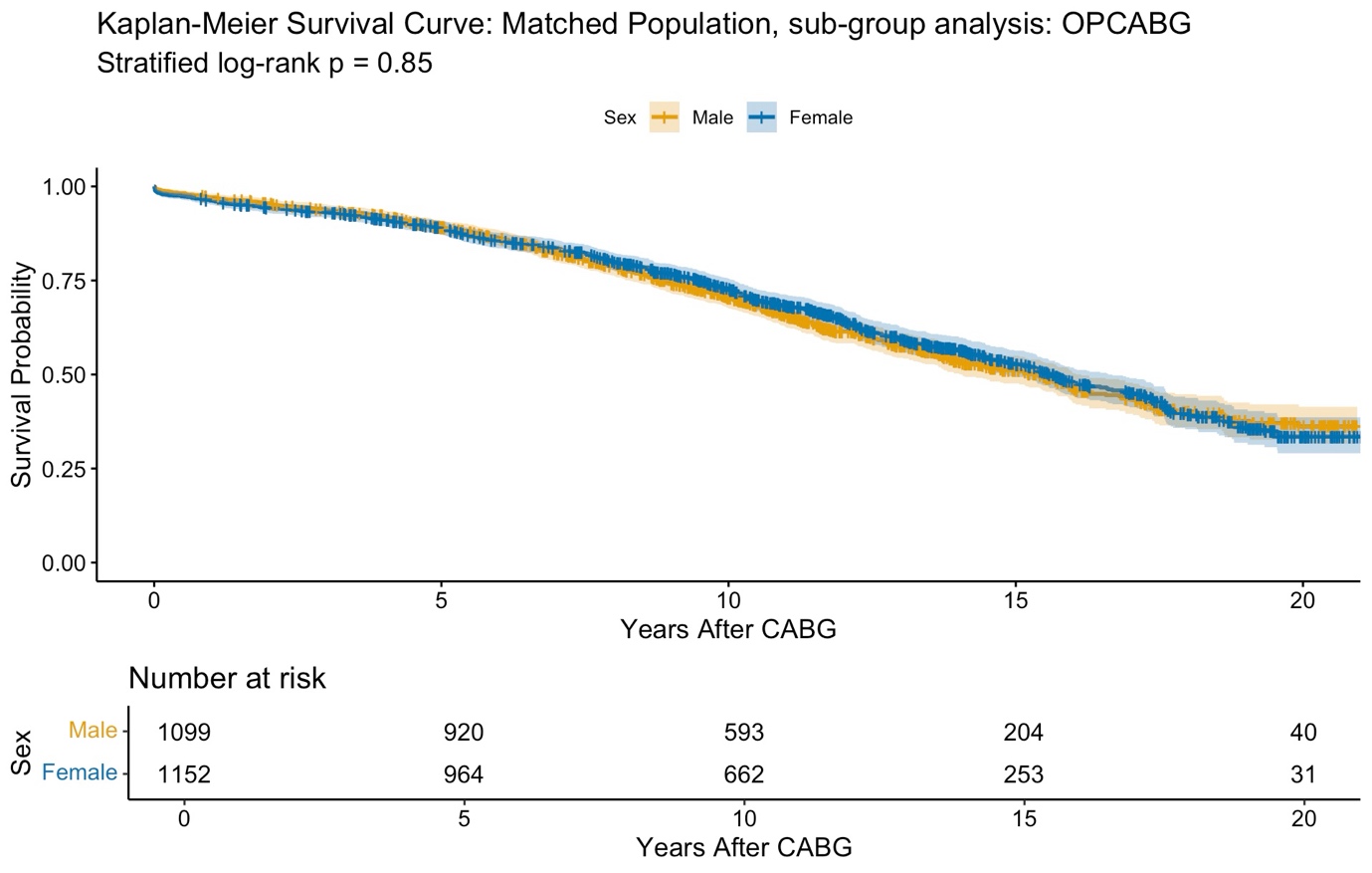


Figure S4: Subgroup analysis: Kaplan-Meier survival analysis of the matched population, by sex, limited to OPCABG
